# Myeloid Sarcoma: Novel Advances Regarding Molecular Pathogenesis, Presentation and Therapeutic Options

**DOI:** 10.3390/jcm13206154

**Published:** 2024-10-16

**Authors:** Michael D. Diamantidis

**Affiliations:** Thalassemia and Sickle Cell Disease Unit, Department of Hematology, 1st Department of Internal Medicine, General Hospital of Larissa, Tsakalov Str. 1, 41 221 Larissa, Greece; diamantidis76@gmail.com; Tel.: +30-2413-504117; Fax: +30-2410-283822

**Keywords:** myeloid sarcoma (MS), isolated MS, synchronous MS, acute myeloid leukemia (AML), extramedullary AML, diagnosis, immunophenotype, molecular background, treatment, induction chemotherapy, allogeneic hematopoietic stem cell transplantation (HSCT), radiotherapy

## Abstract

Myeloid sarcoma (MS), an extramedullary form of acute myeloid leukemia (AML) is a rare tumor mass of myeloid blasts. It can disseminate to any one or multiple anatomical sites, with (synchronous MS) or without (isolated MS) bone marrow (BM) involvement. The aim of this review is to describe the most recent advances in MS regarding diagnosis, molecular background, various clinical manifestations from several organs, and treatment approaches. Due to the lack of prospective, randomized clinical trials, therapeutic decisions are a challenge for the clinician. In the era of novel targeted AML treatments, a critical analysis of how to decide the best option for individual patients, also covering the possible central nervous system (CNS) prophylaxis is provided. For the majority of the patients, AML induction chemotherapy, followed by hematopoietic stem cell transplantation (HSCT) is generally recommended. This paper discusses the role of radiotherapy, the treatment of refractory and relapsed disease, along with the therapeutic approach of difficult-to-treat patients, due to specific problems related to different anatomical sites of MS.

## 1. Introduction—Definitions

Acute Myeloid Leukemia (AML) is an uncommon neoplastic disease, characterized by the presence and accumulation of immature myeloid progenitors in the bone marrow (BM) and peripheral blood (PB). The malignant leukemic blasts expand clonally and show properties of differentiation arrest. However, the term extramedullary AML refers to heterogeneous clinical scenarios of expansion of leukemic blastic cells to tissues and anatomic sites other than the BM and the PB, such as the spleen, liver, lung, mediastinum, pancreas or even the central nervous system (CNS). Almost every organ or tissue has been reported as a site of initial presentation of an extramedullary AML [1,2,3].

Historically, the terms ‘chloroma’, ‘granulocytic sarcoma (GS)’, ‘myeloblastoma’, along with ‘myeloid sarcoma (MS)’ have all been used to describe these extramedullary AMLs. ‘Chloroma’ derives from the Greek word ‘chloros’ meaning ‘green’, because of the green colour of the malignancy, due to the oxidation of myeloperoxidase (MPO) within the granules of immature myeloid precursors [4]. Moreover, sarcomas are tumors of transformed mesenchymal or connective tissue. Mesenchymal tissue arises from the mesoderm during fetal development, giving rise to connective tissue, bone, cartilage, muscle, and vessels. The mesenchymal cell is the stem cell for all connective tissue cells. Furthermore, embryonic mesodermal mesenchyme develops not only into connective tissues but also into blood and blood vessels. During development, hematopoietic stem cells give rise to blood cells, along with endothelial cells, which line blood vessels, both derive from the mesodermal germ-cell layer; exactly how though is debatable. The most probable explanation is that ‘sarcoma’ has prevailed, due to the external morphology that resembles sarcomas. Nevertheless, the true composition of MSs involves myeloid, leukemic blastic cells, located in extramedullary anatomic areas. MS represents a unique tissue-based manifestation of AML [4,5,6,7].

MS may also represent the blastic phase of transformed myeloproliferative neoplasms (MPNs), myelodysplastic syndromes (MDS), or MDS/MPNs. Both 2the 022 International Consensus Classification (ICC) [8] and World Health Organization (WHO) [9] classifications define MS as an ‘umbrella’ of heterogeneous neoplasms, clinically and molecularly, instead of a homogeneous single entity.

MS can occur in an isolated extramedullary form with a normal BM (isolated extramedullary AML, ‘non-leukemic’ or ‘aleukemic’ form), which is usually followed by the development of metachronous intramedullary AML. Nevertheless, MS might also occur in parallel with intramedullary AML, and the term ‘synchronous extramedullary AML’ is used in the latter cases [4].

The incidence of MS at diagnosis, including both synchronous and isolated extramedullary AMLs, varies from 0.2–2.8% [10,11,12,13,14]. However, the presence of extramedullary AML at diagnosis is often underestimated and has been reported to affect approximately 2–8% of newly diagnosed AML cases [11]. Intriguingly, it is increased as an isolated extramedullary AML in the post-allogeneic hematopoietic stem cell transplantation (HSCT) relapse setting, reaching 15% of all post-allogeneic HSCT relapses [11].

Regarding prognosis, MS has a diverse overall survival (OS) varying according to the age, sex, race, and sites of presentation [11]. No prospective studies have been conducted, due to the rarity of MS diagnosis. Hence, data on prognosis are limited and derive from small retrospective studies with known limitations [15]. However, historically, the prognosis in patients with MS has been considered poor for most patients. If an isolated extramedullary MS is left untreated, the patient will eventually develop BM dissemination (AML) within 5–12 months [5,7,16]. The prognosis is also poor when there is CNS involvement [17], or KMT2A rearrangements in pediatric MSs [18]. An abnormal karyotype has also been associated with a statistically significant dismal MS prognosis [19]

Nevertheless, several retrospective studies challenge this notion and report better outcomes [12,13,14,20]. It has been observed that the OS is higher for MSs involving the pelvis, the genitourinary organs, the eyes, the gonads, and the gastrointestinal mucosa in comparison to the nervous system, the soft and the lymphatic and hematopoietic tissues [12]. No statistically significant difference has also been described regarding OS between isolated MS (1-year OS: 60%) and MS with concomitant AML (1-year OS: 50.1%), whereas it was significantly lower for MS after an initial MDS or MPN diagnosis (1-year OS: 14.3%) [20]. Furthermore, the prognosis does not differ when positive T-cell MS cases are compared with negative T-cell MS cases [20]. Randomized trials are essential not only to define prognosis but also to help in clarifying the proper treatment strategy.

## 2. Diagnosis

Patients with MS should be thoroughly investigated, including molecular and cytogenetic studies for guiding appropriate classification and treatment planning. Diagnosis of MS is often challenging and is based mainly on immunohistochemistry, histopathology, and imaging [2,5,6,21,22].

Biopsy of the involved tissue or tissues for multifocal MSs is essential [5]. Core biopsy and fine needle aspiration (FNA) are the applied methods used, each one having its own advantages and disadvantages. Immunohistochemistry (IHC) staining of the dissected tissue by the hollow needles used in core biopsy perseveres the architecture of the tissue, while the rapid, minimally invasive, and low-cost FNA offers good sensitivity and specificity and is more frequently adopted by flow-cytometry based assays for disease immunophenotyping. Moreover, core biopsy provides more specificity than FNA with the additional advantage of ancillary tests [4].

Histological examination of the masses or the tumors shows pleomorphic infiltrate of primitive cells of variable sizes, scattered eosinophilic myelocytes (a useful indication for the diagnosis, albeit not always present), and nuclear configuration with granulocytic and mononucleate cells of variable levels of maturity [5,21]. Four groups based on the degree of maturation have been identified: blastic, immature, differentiated and monoblastic sarcoma [21].

The positivity of several antigens in IHC might vary in MS. MPO is present in 63.2% of all cases, CD13 and CD33 in 48.7%, while CD68 in 51.3% of all patients [20,23]. CD43 is also highly sensitive, although not specific [24]. Other positive antigens with variable incidence are CD117, CD15, CD163, CD34, CD99 and lysozyme [6,23]. Lysozyme, CD68, and CD43 are the markers with the highest sensitivity for MS; yet they are non-specific. Thus, they should not be used as evidence of MS in the absence of CD33, MPO or CD117 [5]. For example, lysozyme is positive for histiocytes, monocytes, and neutrophils and may also stain some epithelial cells. It can be positive in other diseases like histiocytomas, histiocytic sarcomas [25,26] or mastocytosis [27].

Overall, key markers for the diagnosis of MS are MPO, CD43, CD68, and CD117 [5,28]. In a recent publication, 100% of the cases expressed CD43, 85.5% expressed MPO, whereas the positive expression rates of CD117 and CD68 were 62% [28]. The combination of a specific myeloid marker (either MPO or CD117) and CD43 or CD68 poses a strong argument for the diagnosis of MS. The past medical history is important because most patients with MS have a concurrent or antecedent AML [5].

T-cell markers are also expressed (CD5: 34.2%, CD3: 20.7%, CD4), even though all T-cell positive MSs have been reported to be negative for the αβ and γδ T-cell receptors [20,23]. CD7 is positive in a few cases with MS and that may lead to an erroneous diagnosis of T-cell lymphoma. Moreover, CD4 and CD56 are positive in a minority of patients and may lead to a misdiagnosis of T/NK cell lymphoma or a blastic plasmacytoid dendritic cell neoplasm (BPDCN) [5]. A practical algorithm using immunohistochemistry in order to diagnose soft tissue MSs has been proposed [29].

A simultaneous BM biopsy is generally recommended in order to compare the morphologic and immunophenotypic characteristics and to define the extent of the disease by comparison with the flow cytometric analysis of the tissue specimens from various anatomical sites. The prudent interpretation of immunohistochemistry is mandatory to minimize the risk of misdiagnosis [5]. Imaging features on computed tomography (CT) and magnetic resonance imaging (MRI) are also helpful in investigating a patient with MS [22].

Computed tomography (CT) and magnetic resonance imaging (MRI) are helpful in diagnosing MS because MSs present in the form of masses traced by CTs or MRIs. The imaging presentation of MSs is the only initial manifestation in isolated MSs. Moreover, it may precede or occur simultaneously with the systemic hematological disease in some cases of synchronous MS. In most patients with synchronous MS, the imaging presentation occurs after the initial diagnosis of AML. The visceral soft tissue is affected in most cases, followed by the cutis/subcutis, the bones, the CNS, the lymph nodes, and the visceral organs [22].

It is important to note that MSs are usually misdiagnosed as solid tumors. Thus, the respective tissue specimens are maintained in paraffin blocks, which are unsuitable for the analysis of conventional karyotype [15,21,30]. Hence, that explains why cytogenetic data are not available for many patients with MSs, especially with isolated MSs. Consequently, the detection of cytogenetic abnormalities in MSs is usually performed using fluorescence in situ hybridization (FISH) with known limitations related to the quality of the probe and the specimen [2,3,15,21]. A complete screening evaluation for AML-related mutations with next-generation sequencing (NGS) and PCR techniques, easier performed in synchronous MS with assessment of BM involvement, is also necessary in the era of targeted therapies for patients with MSs [30].

## 3. Molecular Pathogenesis

MS may derive from a common or precursor hematopoietic stem cell. The latter can be attributed to the fact that molecular aberrations in MS and concurrent BM disease are concordant in 70% of the patients [9]. Moreover, gene mutations have been found in MS patients with morphologically normal BM, suggesting clonal hematopoiesis (CH) or low-level myeloid clonality in the BM [9]. Coincidently, patients with de novo MS harbor genetic abnormalities like RUNX1-RUNX1T1, CBFB-MYH11, KMT2A-MLLT3, and JAK2 V617F, whereas KMT2A and BCR-ABL1 rearrangements have been observed in secondary cases [31].

Moreover, the vast majority of MS cases with CBFB-MYH11 fusion (94%) show a marked predilection for abdominal sites [32]. In addition, aberrant antigenic expression can be the case in MSs. An example of aberrant expression of myeloid and B-cell markers in an aggressive multiple-site MS has been documented [17]. In the latter case, the neoplastic unique cell population consisted of a ‘hybrid’ of a myeloid lineage precursor cell and of a more mature cell of B-cell lineage. A CNS involvement [vermis, hemispheres of the cerebellum, leptomeninges, and the presence of the leukemic blasts inside the cerebrospinal fluid (CSF)] has been attributed to the aforementioned aberrant antigenic expression [17].

An important aspect highlighting the complex molecular pathogenesis of MSs is the co-existence of T-cell acute lymphoblastic lymphoma (T-LBL) in the lymph nodes with a MS in the pericardium. This patient was diagnosed with abnormal karyotype both to pericardial effusion cells [t(8;21)] and the BM (42,X,-Y,del(2)(p12),-9,-9,del(12)(p11),der(13;17)(q10;q10),add(16)(p13),add(21)(p11)[3]/43,idem,+mar[8]/45,idem,-10,+15,+18,+20,+mar[4]) and had multiple serous effusions [33]. In addition, TET2, AML1, and TP53 mutations were identified in the pericardial effusion cells and the BM cells, forming the hypothesis that these mutations may participate in a common pathway to the pathogenesis of T-LBL and MS [33].

The most common of cytogenetic abnormalities encountered in patients with MS are found at the site of extramedullary oncogenesis and are the following: t(8;21), inv(16), t(9;11), del(16q), t(8;17), t(8;16), t(1;11), t(6;10) and aberrations of chromosomes 4, 7, 8, X, Y and 11 [5,7,23,34]. Importantly, t(8;21) is often observed in orbital MS (the most frequent localization of MS in children) of childhood leukemia, whereas inv(16) is more commonly found in gastrointestinal MS [15,30,34,35].

NGS in combination with the contribution of other more conventional molecular techniques like polymerase chain reaction (PCR) for genes of very large size has identified a vast spectrum of mutations in patients with MS [30,36]. The most common mutations are internal tandem duplication of FMS-like tyrosine kinase 3 (FLT3-ITD, 15% of all MS patients, FLT3-TKD), nucleophosmin 1 (NPM1, 15–28%), isocitrate dehydrogenases (IDH: IDH1R132 in 26%, IDH2R140 in 7–11% up to 29% in isolated MSs, IDH2R140Q), receptor tyrosine kinase (c-KIT D816V), DNA methyltransferase 3a (DNMT3A), neuroblastoma-RAS (NRAS), Kirsten-RAS (KRAS), runt-related transcription factor 1 (RUNX1), Casitas B-lineage lymphoma (CBL), V-Raf Murine Sarcoma Viral Oncogene Homolog B (BRAF V600E), PTPN11, CSF3R, SFRP1, SETBP1 and TP53 (21%) [15,23,34,36,37,38,39,40].

The mutated genes in MS include tyrosine kinases (FLT3 and c-KIT), epigenetic modifiers (TET2, ASXL1 and EZH2), spliceosome proteins (SF3B1, SRSF2), cohesins (STAG2), transcription factors (GATA2, PHF6) or tumor suppressors (WT1, TP53) [23,38,40]. In most patients with MS, two mutations are present, the most frequent being NPM1, NRAS, and DNMT3A [36,38]. In addition, multiple mutations were observed in the same patients, proving that a single mutation is not sufficient to cause malignant transformation [36,40]. Finally, PD-L1 expression is increased in 10–12% of MS cells and 37.8% of stromal cells in the tumor microenvironment [30].

Except for MS, leukemia cutis (LC) represents a well-established extramedullary manifestation of AML. LC is considered a type of MS with skin leukemic dissemination. Novel mutations and clones arise during and after the course of treatment, providing molecular insight to the mechanisms of leukemic relapse and resistance to drugs. Hence, a patient with a refractory AML, harbored RUNX1, IDH1, and DNMT3A mutations at diagnosis. After multiple chemotherapies, the patient lacked RUNX1 mutations. However, the novel presentation of LC became evident. LC exhibited novel NRAS mutations detected through NGS examination of the skin lesions in addition to the previous IDH1 and DNMT3A clones from the initial diagnosis [41]. After subsequent courses of treatment, new LC clones appeared in the BM harboring the novel NRAS mutations, establishing the clonal evolution. The latter indicates the acquisition of new mutations from extramedullary leukemia (LC), which became the dominant clone guiding the relapse and the resistance to previous treatment [venetoclax (VEN) plus azacitidine (AZA)] [41]. As a conclusion, gene mutations at the site of presentation of MS are not always identical with those present in the BM [30,42]. Such patients usually have poor overall survival (OS) [30,42].

Specific genetic abnormalities increase the tendency of MS cells to make their home outside of the BM. This predilection for particular anatomic sites might be determined by a complex interplay of factors, such as the expression of chemokine receptors and adhesion molecules (CD56), controlled by epigenetic and genetic mechanisms [40]. The expression of CD56 might increase the ability of blastic cells to circulate and home to the extramedullary sites [21]. Nevertheless, CD56 overexpression has not been documented in most cases of MS. In addition, the rate of CD56-positive leukemic cells is similar in patients with and without MS [2]. Understanding the biological differences among various anatomic sites of presentation is intriguing.

The surface protein CD11b (surface β2-integrin member macrophage-1 antigen) has been implicated in MS development. However, despite the fact that it is selectively expressed in mononuclear cells, a direct causal link between the antigen and leukemic infiltration of sites has not been established. These findings simply describe the enrichment of monoblastic or myelomonocytic MS blastic cells phenotype with CD11b expression [43]. Furthermore, leukemia cutis cells express the following set of receptors (CCR5, CXCR4, CXCR7, and CX3CR1) [44]. Interactions with epithelial CXL12, which is a ligand for CXCR7 and CXCR4, might contribute to the development of MS [45].

It has recently been established that a complement C1Q positive macrophage-like leukemic subset is enriched within the MS and pre-exists in the BM. Hence, C1Q expression, regulated by the transcription factor MAF BZIP transcription factor B, provided leukemic cells with tissue infiltration capacity. The aforementioned study was conducted using single-cell analysis (RNA sequencing both on BM and MS from leukemia cutis samples) [46].

Many similarities exist regarding the epigenetic microRNA profiling, investigated in skin biopsies, between MS and BPDCN, representing evidence of the relevance between the two neoplasms at the molecular level [47]. MicroRNAs are small noncoding molecules serving as regulators of post-transcriptional control of gene translation. Despite the overall similarity between MS and BPDCN, 6.4% of the studied microRNAs are documented to be differentially expressed between the two types [47].

Table 1 summarizes the molecular markers and cytogenetic abnormalities of patients with MSs in relation to clinical manifestations.

## 4. Clinical Studies and Manifestations

Several series of cases involving MSs have been documented, either de novo or secondary due to other neoplasms (of myeloid origin or not) [31]. In the majority of the cases, de novo MSs are present as isolated tumors and mainly have core-binding factor (CBF) genetic rearrangements, whereas secondary MSs have frequently been associated with a complex karyotype. The most common localizations are the skin and the lymph nodes, while the most frequent histologic subtype is monocytic [31,48]. A fatal disseminated skin de novo MS with a wide spread in a 17-year-old male patient has been described [49]. Coincidently, therapy-related MSs (t-MSs) with particular clinicopathological and molecular features following prior cytotoxic therapy and/or radiation have been described [50].

Testicular dissemination is statistically more frequent in MS as an AML recurrence than in other types of MS [20]. In general, the testes are sanctuaries allowing survival of leukemic cells, due to poor penetration by antileukemic drugs. An incidence of 5.8% at a median age of 42 years has been reported for MSs involving the reproductive system overall [11]. In one of the largest series until today, 68 patients diagnosed with testicular myeloid sarcoma (TMS) with a median age of 41 years have been reported [51]. Patients with TMS are diagnosed at least a decade earlier, compared to the median age of diagnosis for all cases of MS (59 years) [11].

Monocytic morphology (FAB M4 and M5), t(8;21), inv(16) and 11q23 cytogenetic aberrations are predisposing risk factors for the development of TMS [52]. In most cases, TMS presents on the left side as a unilateral disease. Common presenting features are testicular or scrotal swelling with pain or not or masses. However, atypical sites and presentations of TMS are often (hydrocele, testicular torsion, Fournier gangrene), making some cases very hard not only to diagnose but even to grow a degree of suspicion for TMS. The latter has also been detected incidentally during an evaluation for left arm paresis, blurring of vision, and an ileal mass. Hence, an incorrect diagnosis of lymphoma, plasmacytoma, or epididymitis, instead of the correct TMS, is common [51].

Intriguingly, 55 cases of gynecological MS have been identified in the Chinese population within a period of twenty years. Most patients showed involvement of the uterine cervix, followed by the ovary and the vulva in descending incidence. The onset of ovarian MS has been reported to a much earlier age than the other sites. Vulvar MS exhibited a high rate of synchronous and secondary AML within a short time of its occurrence [53]. A rare MS with KMT2A (MLL)-ELL fusion presenting as a vaginal wall mass has also been observed in a 53-year-old female patient with a history of vaginal bleeding and uterine fibroids [54]. This patient also harbored losses of chromosomes 1p, 9, 10, 15, 18, gain of chromosome 1q and PTPN11 and FLT3-ITD mutations [54]. MSs of the breast have also been documented as a relapse of AML even after HSCT [55]. Some MSs involving the breast harbor MLL-AF9 rearrangements [56]. Many of the breast MSs were initially misdiagnosed as malignant melanomas, breast carcinomas, or lymphomas [57].

The heart is another site for locating cardiac MSs, which can present with a wide spectrum of clinical symptoms, such as dyspnoea on exertion, chest pain, arrhythmias, tachycardia, palpitations, or heart block. Pericarditis is also a frequent presentation with the presence or absence of leukemic cells in the pericardial fluid. A case series of 30 patients diagnosed with cardiac MS (CMS) has been reported [58].

MS involving the head and the neck region shows a predilection for the oral cavity, especially the gingiva, followed by the palate and the buccal mucosa [24,59]. Patients present with non-specific clinical manifestations, like jaw pain, sore throat, sinus pain, tonsillar enlargement, skin lesions (rash, nodules, papules), or lymphadenopathy. The diagnosis is often challenging because MS of the oral cavity might mimic a lymphoma, a plasmacytoma, an abscess, a pyogenic granuloma, or other inflammatory diseases (chronic gingivitis), thereby delaying the proper diagnosis [24]. BPDCN, solid tumors like melanoma and carcinoma, and other tumors complete the full differential diagnosis [24,59].

MS can also be diagnosed in several sites of the gastrointestinal tract. A total of 17 patients diagnosed with gallbladder MS (GB-MS) have been studied with a median age of 52 years (range 23–84 years) [60]. The initial symptoms vary and include abdominal pain, jaundice, weight loss, vomiting, melena, abdominal distension, palpable mass, anorexia, fever, malaise, and back pain. In all cases, non-specific symptoms of cholecystitis were present. The prognosis of GB-MS is considered poor in general [60]. An interesting case of MS of the bile ducts presenting as obstructive jaundice and initially wrongly considered as a cholangiocarcinoma has been reported [61].

The pancreas is an organ of the presentation of MSs with a dismal prognosis. An isolated pancreatic MS is an extremely rare entity and there are few cases of simultaneous localization of MS in other organs, along with the pancreas [17]. Most cases involve the head of the pancreas, whereas presentation of MS in the pancreatic tail, causing splenic infarction has been observed [62]. Acute epigastric pain, obstructive jaundice, weight loss, and gastrointestinal bleeding are among the clinical manifestations of pancreatic MSs [62,63,64,65].

Several organs can be involved in the dissemination of a MS. Intriguingly a MS involving the small intestine, the mesentery, the mesenteric lymph nodes, and the kidneys have been observed [66]. In particular, renal MSs have been very rarely described in the literature [66,67]. A ureteral MS after renal transplantation, along with acute promyelocytic leukemia (APL) has been reported (synchronous MS) [68]. Other examples of combinations of organs involved in a diagnosis of MS are the gastric antrum, the duodenum, the gallbladder, and the pancreatic head in one female patient [69] or the manubrium of the sternum, the lumbar vertebrae, the sacrum, the pancreatic head, and the duodenum in another [17].

Moreover, a late relapse of AML after 9 years of the initial diagnosis and treatment as MS-causing radiculopathy has also been documented [70]. Several masses in the cerebellopontine angle projecting towards the internal auditory canal and at the levels of C7-T1 were diagnosed as a multi-focal MS. Thickening in the dorsal radix of C7-C8 and multiple intrathecal masses between L1-S1 were also observed [70]. The patient suffered from left-sided hearing loss, tinnitus, and difficulty in swallowing and was initially diagnosed as having a schwannoma. The correct diagnosis of MS as a late AML relapse was made following CSF cytological and immunophenotypic examination after the patient had progressive muscle weakness in both legs, paresthesia, nystagmus at both lateral gaze, and final paraplegia [70]. The patient received intrathecal, systemic chemotherapy and radiotherapy and experienced full clinical and neurological recovery [70].

Isolated localizations of MS have been described in the pancreas [64,65], in the large bowel [71] (right colon [71]), in the small intestine and the mesentery [72], the anal fissure [73], the peritoneum (after treatment for a testicular seminoma) [74], the placenta [75], the lung [76], the conjunctiva [77], the corneal limbus of the eye (limbal mass) [78,79], the external auditory canal bilaterally [80], the left cerebellar hemisphere (intracranial) [81], the scalp [82], the femur (pathological fracture) [83], the skin (aleukemic cutaneous MS) [84], the parotid [85], the jaw, facial nerves, lips, nasal cavity [21,24] and the gingiva (oral and maxillofacial cases) [86]. Surprisingly, the absence of AML progression has been observed in a female patient with 2 collision tumors (MS and concomitant right colon adenocarcinoma), who was treated for the large bowel neoplasm [71].

In the interesting case of the 34-year-old pregnant woman diagnosed with mixed-lineage leukemia rearranged AML during the third semester, delivery of a healthy newborn was performed. Then, induction chemotherapy was applied without achievement of disease remission. After evaluation of the placenta, a focal nodule was observed. The diagnosis of placental MS was established after a pathologic examination of the specimen. Luckily, the infant remains healthy with normal blood counts after close follow-up. Transplacental transmission of leukemic blasts cannot be excluded in the rare cases of maternal AMLs [75].

A concurrent presentation of MS (lymph node) and a monoclonal kappa CLL/SLL (BM) for the first time in the same patient, a combination of two distinct neoplasms in different sites has been described [87]. Moreover, the simultaneous occurrence of a penile MS and of a BM B-cell neoplasm (multiple myeloma) has also been documented [88]. Another interesting association is the co-existence of a mixed phenotype (myeloid/B-cell) acute leukemia (MPAL) with a MS of the thyroid gland [89]. MSs of the thyroid gland are extremely rare. Additionally, MPAL in association with MS has been described very few times before [89].

A link between MPNs and MSs has been established. An extramedullary blastic transformation of primary myelofibrosis in the form of a disseminated MS has been documented [90]. Patients with chronic myeloid leukemia might develop a cutaneous MS [91] or a MS even after complete hematological, cytogenetic, and molecular remission [92].

Table 2 provides an overall summary of the number of cases reported at various anatomic sites. The most important studies involving case series, along with a literature review were included in the table, whereas isolated cases were reported inside the text in detail.

## 5. Treatment

Due to the rarity and heterogeneity of MSs, there is a lack of prospective randomized clinical trials. Choosing the proper therapeutic strategy is a complex and multifactorial decision taking into account the characteristics of the patient (age, performance status, comorbidities, personal wishes), the disease biology (karyotype, mutational profile) and the availability of the donors, in case of allogeneic HSCT. Other important elements include the anatomic site and the size of MS, the existence of isolated vs. synchronous MS, the presence of de novo or relapsed or refractory disease, or even the possibility of relapse after allogeneic HSCT. Since all diagnosed patients with MS will inevitably develop AML after a time interval, treatment with systemic AML protocols should be applied if feasible, even in isolated MS. The role and type of consolidation therapy have yet to be defined. Local therapies (radiotherapy, surgery), systemic chemotherapy, HSCT for fit patients, targeted therapies, immunotherapies, and the possible administration of CNS prophylaxis for all or selected patients should be under thorough consideration and debate [3,4,5,15,16].

The role of surgery should be restricted to diagnostic purposes, as surgical excision biopsy may be useful in obtaining tissue samples for the correct diagnosis. On the other hand, involved field radiotherapy should be considered for all patients with isolated MSs and it is recommended for all patients with de novo MS, who are refractory to systemic therapy or with isolated recurrence after HSCT [93,94]. Moreover, radiotherapy is proposed for palliation of symptomatic vital structure compression (spinal cord extramedullary AML). The potential need for future total body irradiation (TBI), as part of the conditioning strategy, if the patient becomes a candidate for allogeneic HSCT should also be considered [4].

Concerning the radiation dose, a range of doses of 10–30 Gy over 1–3 weeks can be effective [95]. Another proposed regimen is 24 Gy in 12 fractions. Lower doses (6–20 Gy) can be administered when the condition of the patient does not allow a more protracted course [94]. For symptomatic leukemia cutis, a similar dose regimen can be applied for isolated skin lesions. For persistent diffuse, disseminated leukemia cutis with a lack of BM involvement, total skin electron beam therapy to a dose of up to 24 Gy can be considered [94]. Additionally, craniospinal radiotherapy has been proposed for CNS dissemination of the MSs, despite the lack of solid data from prospective randomized clinical trials [17,96]. Finally, no benefit has been shown from combination therapy of radiotherapy in addition to systemic chemotherapy for extramedullary AML in general [97].

Most patients with MS will progress to AML within 4–6 months if left untreated or treated with local therapies (radiation, surgery). Thus, induction AML chemotherapy is essential with anthracycline and cytarabine-based regimens for fit patients (7 + 3 regimen), even in isolated MSs, as supported by NCCN guidelines [2,3,4,15,16,93,98]. However, ELN 2022 recommendations offer no specific guidelines for treating MSs [99]. No MS-specific treatment regimens have been adopted. CR rates of 69% in patients with isolated MS treated with cytarabine, idarubicin, and fludarabine [13] For patients unfit for intensive chemotherapy, hypomethylating agents (azacitidine, decitabine), alone or more recently in combination with venetoclax have been administered [2,100,101,102].

The treatment landscape of AML (which consequently affects the therapy of MS) has radically changed in the last few years with the approval of novel targeted therapies. It is a challenge for the clinician because a better evaluation and understanding of the molecular background of the disease is mandatory in order to provide the best possible treatment options for the patients. Such examples are the following novel drugs or therapies: ivosidenib, enasidenib, and olutasidenib (IDH inhibitors), FLT3 kinase inhibitors (midostaurin, gilteritinib, quizartinib, and sorafenib), glasdegib (Hedgehog signaling pathway), CPX-351 (an intensive chemotherapy regimen consisting of cytarabine and daunorubicin in the liposomal form at a fixed 5:1 ratio), along with the BCL-2 inhibitor venetoclax [30,103,104,105]. Very few data exist in the literature regarding efficacy and side effects of these drugs in patients with MS and they mainly are isolated case reports.

Treatment with ivosidenib (for patients with IDH1 mutations) or enasidenib (targeting IDH2 mutations) led to complete remission (CR) with a median response duration of 15 months in a retrospective study of 58 IDH mutated MS patients [106]. Furthermore, isolated published cases documenting efficacy of the FLT3 inhibitor gilteritinib in FLT3 mutated MSs have been described [36,79,107,108,109,110].

A difficult task for clinicians is to treat patients with a CNS dissemination of MSs, due to the lack of prospective, randomized clinical studies. For MS patients who have neurological manifestations in association with leptomeningeal disease, radiotherapy with intrathecal chemotherapy (methotrexate plus cytarabine plus hydrocortisone or thiotepa for leptomeningeal involvement) and/or systemic chemotherapy (high-dose cytarabine or high-dose methotrexate) have been proposed [15,16,17,87,94,96,111,112]. Systemic chemotherapy serves as an adjunct to intrathecal treatment and it is generally avoided for two weeks before, concurrently, and for two weeks after cranial radiotherapy. The addition of novel targeted therapies, such as gilteritinib to systemic chemotherapy, for FLT3-positive patients, can be effective [108]. Sorafenib in combination with conventional chemotherapy has shown promising results in refractory FLT3-ITD mutated AML patients with CNS dissemination (CR: 81%, overall response rate: 89%, 2-year event-free survival: 75%, 2-year OS: 77%) [113]. Interestingly, venetoclax can pass through the blood-brain barrier and can be effective in MSs with CNS involvement [114].

CNS Prophylaxis in patients with MS in order to prevent CNS dissemination of the neoplastic leukemic cells is another topic of debate. No data from randomized clinical trials exist until today and decisions depend on the protocols applied to individual centers. The number of cycles and the type of prophylaxis are also controversial issues, due to the potential toxicity. Some centers do not provide CNS prophylaxis for adults with MS or extramedullary AML who have no neurologic manifestations or other evidence of CNS involvement, because there is no proven benefit and it avoids potential adverse effects of prophylaxis. However, CNS prophylaxis is routinely administered for patients with BPDCN, a neoplasm sharing common molecular defects with MS, making the subject even more complicated [47,111,112].

In addition, data from isolated cases have linked the monoblastic or myelomonocytic element of MSs, the aberrant immunophenotypic antigenic expression of myeloid and B- or T-cell markers, along with the involvement of multiple anatomic sites as predisposing risk factors for CNS dissemination of MSs, rendering prophylactic treatment essential in such patients [17,79,87,96,108,113]. Other studies consider young age (some define younger age < 64 years), elevated LDH at presentation, increased white blood cell count, trisomy 8, FLT3-ITD mutations, 11q23 abnormalities, a prominent monocytic component and core-binding factor (CBF) AMLs, like t(8;21) or inv(16) as factors associated with an increased risk of AML involvement in the CNS [111,115,116,117,118]. Nevertheless, the successful application of high-dose cytarabine in CBF AML might moderate the risk of CNS dissemination. Therefore, the protocols of some centers have incorporated 2–4 cycles of intrathecal CNS prophylaxis even in isolated MSs with an increase of the cycles for patients having more predisposing risk factors, as previously defined.

Solid data or prospective randomized studies supporting the application of allogeneic HSCT in first remission as a consolidation strategy for patients with isolated (low patient number, heterogeneity of presentation) or synchronous MS are lacking. The decision to proceed to allogeneic HSCT in isolated MS in the first CR should be made cautiously for each patient, due to the morbidity and the associated complications of transplantation, like graft versus host disease (GVHD) [15]. It has been reported that T-cell-depleted grafts, reduced intensity conditioning (RIC) regimens, or non-TBI-based conditioning regimens are associated with higher incidences of MS relapse after allogeneic HSCT [119,120,121]. The 5-year OS post allogeneic HSCT for patients with extramedullary AML ranges from 47 to 53% [98,122,123,124]. Younger age, CR prior to transplantation, and the use of myeloablative conditioning have been correlated with a better outcome [124]. Finally, pediatric patients with AML and MS may not benefit from HSCT [18].

However, no impact of pre-transplant extramedullary disease or MS on the outcome of allogeneic HSCT has been documented in a large study including 310 centers from 44 countries [125]. No impact between non-myeloablative and myeloablative (including TBI) conditioning on the possibility of relapse has been demonstrated [125]. In conclusion, high-risk patients with an intermediate or adverse prognosis, based on cytogenetic and molecular data, should undergo allogeneic HSCT in the first CR, if possible. Allogeneic HSCT should also be offered in cases of relapsed or refractory MS. Consolidation without allogeneic HSCT, but only with chemotherapy should be reserved for unfit patients for transplantation or for patients with low-risk disease [2,3,4,15,16]. The selection of the type of conditioning regimen depends on the protocols and the experience of the individual center, the comorbidities and the patient’s medical history, and the availability of TBI [2,4,126].

Treating relapsed MS remains problematic, due to the aggressiveness of the disease at this stage (median OS of relapsed patients following allogeneic HSCT: 4–6 months) [15]. Apart from radiotherapy and a second HCST if possible, other strategies include donor lymphocyte infusion, systemic salvage chemotherapy with agents not received before, or the administration of hypomethylating agents, alone or in combination with venetoclax [3,4,15]. Immunotherapy with the anti-CTLA4 monoclonal antibody (Ipilimumab) demonstrated CR for all patients with extramedullary AML, who had relapsed after allogeneic HSCT [127].

Especially for TMS, the combination of chemoradiotherapy followed by HSCT has shown the best outcome [51]. Moreover, for vulvar and gynecological MS, the disease should be treated aggressively, despite its limited distribution, with chemotherapy followed by allogeneic HSCT, upon the availability of the appropriate donor [53]. Furthermore, CMS carries a dismal prognosis despite various chemotherapeutic and radiation treatment protocols. It is well known that anthracycline-based regimens cause cardiotoxicity. Therefore, such protocols cannot be applied to the treatment of CMS [58].

## 6. Scientific GAPS—Current and Future Research

The molecular events (mutations and cytogenetic abnormalities) allowing some AMLs to have a predilection for extramedullary sites are unknown until today, despite the progress and a better understanding of the molecular background of MSs [5,30]. The reasons why some AMLs manifest originally as extramedullary diseases (isolated MSs) have not been fully elucidated. Furthermore, the molecular relationship of MSs to the BM disease is an area of uncertainty. Finally, the question of whether the MS represents a clonal evolution of the original AML or not remains to be answered [5].

With the advent of molecular techniques (NGS) and the expansion of targeted therapies, there is significant potential for improvement in the field of refractory MS patients. Further research is necessary to detect novel molecular markers for diagnosis, standardize diagnostic methods, and establish effective chemotherapy regimens and radiation protocols for MS patients. Such cases are very rare and that is the difficulty in designing prospective randomized clinical trials. Only through well-designed clinical trials can evidence-based treatment decisions be made for MSs.

## Figures and Tables

**Table 1:** Molecular markers and cytogenetic abnormalities of patients with Myeloid Sarcomas (MSs) in relation to clinical manifestations.

Molecular Markers or Cytogenetic Abnormalities of MSs	Clinical Characteristics of MSs or Outcome
Core Binding Factor (CBF) Rearrangements	De novo myeloid sarcomas (MSs)
CD56	Higher incidence of MS, extramedullary dissemination, AML with t(8;21)
Complex Karyotype	Secondary myeloid sarcomas
CXCR4 increased expression	Poor prognostic factor in MS
t(8;21)	Orbital MS of childhood leukemia
inv(16)	Gastrointestinal MS
Monocytic morphology (FAB M4-M5), t(8;21), inv(16), 11q23	Predisposing risk factors for testicular MS
MS with CBFB-MYH11 Fusion [inv(16)] or t(16;16)	Abdominal dissemination—involvement
Aberrant myeloid and B-cell or T-cell antigenic expression, multiple extramedullary anatomic sites, young age, age < 64 years, elevated LDH, increased WBCs, trisomy 8, 11q23 abnormalities, a prominent monocytic component, CBF AMLs [t(8;21), inv(16)]	Involvement of the central nervous system in MS
Complex Karyotype, TET2, AML1, TP53 mutations	Co-existence of MS and T-LBL
MLL-AF9 rearrangement	Breast localization of MSs
KMT2A (MLL)-ELL fusion, complex karyotype, PTPN11 and FLT3-ITD mutations	Vaginal MSs
FLT3-ITD mutations in MSs	Central nervous system (CNS), breast, lung, and soft tissue involvement
IDH2 mutant MSs	Subcutaneous and intracranial dissemination
Increased C1Q expression	Tissue infiltration capacity for leukemic cells causing MS

**Table 2:** The most important studies with cases of myeloid sarcomas. CNS: Central Nervous System, MS: Myeloid Sarcoma, OS: Overall Survival.

Number of Patients	Anatomical Sites Involved	Outcome	Comments	References
131 patients	Lymph Node (72), Skin (29), Mediastinum (28), Testis (8), Laryngopharynx (7), Pleural—Abdominal Cavity (7), Oral Cavity (4), Orbit (4), Lung (4), Gastrointestine—Colon (3), Uterine (3), Bone (3), CNS (2), Bladder (2), Thyroid (1), Breast (1), Pancreas (1), Kidney (1)	1-year-OS: Isolated MS (60% alive), Synchronous MS (50.1% alive), MS after MPN/MDS (only 14.3% alive)	Some patients have disease in multiple sites (more than 1 organ)	Kawamoto K et al., 2016 [20]
2. 68 patients	Testis (68)	Disease-related deaths (47%), Alive in remission (33%), Alive—Active Disease (11%), Non-disease related deaths (5.4%)	Lost to follow-up (19%)	Sahu KK et al., 2019 [51]
3. 55 patients (Gynecological MS)	Uterine cervix (22), Ovary (13), Multifocal (9), Vulva (6), Uterine body (3), Vagina (2)	Disease-related deaths (22%)	Vulvar and multifocal MSs with the poorer prognosis	Zhang X et al., 2019 [53]
4. 32 patients	Pleural effusion (2), Multifocal (9), Eye (2), Lymph nodes (7), Soft tissue (3), Uterus (1), Pelvic mass (1), Breast (1), Femur (1), Kidney bladder (1), Abdominal mass (1), Humerus (1), Pericardium (1), Renal mass (1)	Median OS: 4 months (range 0.3–96 months)	Lost to follow-up (5/32, 16%)	Paydas S et al., 2006 [67]
5. 30 patients	Cardiac (30)	Disease-related deaths (14/30, 47%), Alive in remission (9/30, 30%), Alive—Active Disease (3/30, 10%)	Lost to follow-up (4/30, 13%)	Gautam A et al., 2017 [58]
6. 17 patients (Head—Neck MS)	Tongue (1), Tonsils (3), Lymph nodes (1), Skin of cheek or eyelid (3), Gingiva (3), Lip (2), Mandible (1), Buccal soft tissue (1), Turbinate (1), Maxillary Ridge (1)	Disease-related deaths (10/17, 59%), Alive (5/17, 29%),	Lost to follow-up (2/17, 12%)	Zhou J et al., 2013 [24]
7. 17 patients	Gall bladder (17)	Disease-related deaths (56%)	3 cases initially misdiagnosed as lymphoma	Sahu KK et al., 2020 [60]
8. 8 patients	Pancreas (8)	Disease-related deaths (2/8, 25%), Alive in remission (4/8, 50%), Non-disease related deaths (1/8, 12.5%)	Lost to follow-up (1/8, 12.5%)	Li XP et al., 2011 [62]
